# *Bartonella* spp. in Bats, Kenya

**DOI:** 10.3201/eid1612.100601

**Published:** 2010-12

**Authors:** Michael Kosoy, Ying Bai, Tarah Lynch, Ivan V. Kuzmin, Michael Niezgoda, Richard Franka, Bernard Agwanda, Robert F. Breiman, Charles E. Rupprecht

**Affiliations:** Author affiliations: Centers for Disease Control and Prevention, Fort Collins, Colorado, USA (M. Kosoy, Y. Bai, T. Lynch);; Centers for Disease Control and Prevention, Atlanta, Georgia, USA (I.V. Kuzmin, M. Niezgoda, R. Franka, C.E. Rupprecht);; National Museum of Kenya, Nairobi, Kenya (B. Agwanda);; Centers for Disease Control and Prevention in Kenya, Nairobi (R.F. Breiman)

**Keywords:** Bacteria, Bartonella, bats, zoonoses, Kenya, research

## Abstract

We report the presence and diversity of *Bartonella* spp. in bats of 13 insectivorous and frugivorous species collected from various locations across Kenya. *Bartonella* isolates were obtained from 23 *Eidolon helvum,* 22 *Rousettus aegyptiacus,* 4 *Coleura afra*, 7 *Triaenops persicus*, 1 *Hipposideros commersoni*, and 49 *Miniopterus* spp. bats. Sequence analysis of the citrate synthase gene from the obtained isolates showed a wide assortment of *Bartonella* strains. Phylogenetically, isolates clustered in specific host bat species. All isolates from *R. aegyptiacus, C. afra,* and *T. persicus* bats clustered in separate monophyletic groups. In contrast, *E. helvum* and *Miniopterus* spp. bats harbored strains that clustered in several groups. Further investigation is needed to determine whether these agents are responsible for human illnesses in the region.

An unprecedented, increasing interest in bats as reservoirs of infectious diseases occurred during the past decade. Mounting evidence indicates an association of bats with various emerging infections, some with high mortality rates, including lyssaviruses, severe acute respiratory syndrome and other coronaviruses, and henipa, Ebola, and Marburg viruses (*1**–**6*). However, the list of pathogens discovered in bats is even more extensive and includes other representatives from various taxonomic groups (*6**–**8*). Most infectious agents in bats are viruses; bacterial species have been rarely reported (*9*). Excluding reports resulting from serologic and microscopic observations before the 1990s, only a few recent publications describe bacterial species in bats. One publication reports fatal borreliosis in a bat caused by a relapsing fever spirochete in the United Kingdom (*10*). Another study on the molecular detection of hemoparasites infecting bats in southwest England showed the presence of *Bartonella* spp. DNA in the blood of 5 of 60 tested bats (*11*). This report on bat infection potentially caused by bacteria of the genus *Bartonella* is consistent with studies showing detection of *Bartonella* spp.–specific DNA in ectoparasites collected from bats in Egypt and the United States (*12**–**14*).

*Bartonella* spp. are mainly hemotropic, facultative intracellular parasites associated with erythrocytes and endothelial cells of mammals (*15**,**16*). *Bartonella* spp. organisms are highly adapted to a wide variety of mammals, including rodents, insectivores, carnivores, ungulates, and marine mammals such as dolphins. New insights into the natural history of various *Bartonella* spp. suggest that these bacteria have adapted to their mammalian reservoir hosts in unique ways with frequently restricted host species ranges (*17*). The bacteria can cause chronic intraerythrocyte infections that sometimes result in a large proportion of the reservoir host population being bacteremic simultaneously (*18*). Infections usually cause few or no clinical signs in the reservoir hosts. Host adaptation is evident as some *Bartonella* species and genotypes are found in very specific mammalian species.

Available data on *Bartonella* spp. have expanded rapidly during recent years, as this group of organisms has been found to be associated with a growing spectrum of emerging and reemerging diseases. In addition to cat-scratch disease, trench fever, and Carrión disease, other illnesses linked to *Bartonella* spp. infection range from a self-limiting, short-term fever to potentially fatal systemic diseases with cardiovascular, nervous system, or hepatosplenic involvement (*19*). Some *Bartonella* spp. that have been implicated as human pathogens are linked to rodent species; these species include *B. elizabethae*, *B. grahamii*, *B. washoensis*, and *B. vinsonii* subsp. *arupensis* (*20**–**23*). Other *Bartonella* spp. are linked to wild and pet carnivores. For example, *B. henselae*, carried by cats, causes cat-scratch disease in immunocompetent persons and bacillary angiomatosis in immunocompromised persons (*19**,**24*); *B. vinsonii* subsp. *berkhoffii* has been carried by dogs and is responsible for endocarditis in a human patient (*24**,**25*). For some *Bartonella* spp. recently implicated as human pathogens (such as *B. rochalimae*, which was isolated from an American tourist traveling to Peru, or *B. tamiae*, isolated from 3 patients in Thailand), a mammalian reservoir has not been determined despite a wide range of tested animals collected in these countries (*26**,**27*). These unidentified reservoirs indicate a need for extensive surveillance among diverse groups of animals for *Bartonella* strains, especially among bats, which represent around 20% of all mammalian species (*6*).

This study was conducted within the framework of the Centers for Disease Control and Prevention (CDC) Global Disease Detection program, which is designed to estimate the health and financial effects and transmission patterns of emerging infectious pathogens associated with humans and animals (including bats, rodents, and other likely reservoirs for human infection) in Kenya and other locations. This study had 5 objectives: 1) to estimate prevalence of *Bartonella* spp. infections among diverse chiropteran species in Kenya; 2) to isolate and identify detected *Bartonella* spp. and create a reference collection of *Bartonella* isolates from East Africa with further characterization and diagnostic investigation; 3) to evaluate genetic heterogeneity of circulating *Bartonella* strains by using the partial sequence variability of the citrate synthase gene (*gltA*), proven to be an excellent genetic marker for analysis of *Bartonella* strains (*28*); 4) to compare *Bartonella* strains obtained from bats in this study with strains obtained from other animal reservoirs and available from the public domain; and 5) potentially to identify new species of *Bartonella*.

## Material and Methods

### Blood Samples Collection

**Figure 1:**
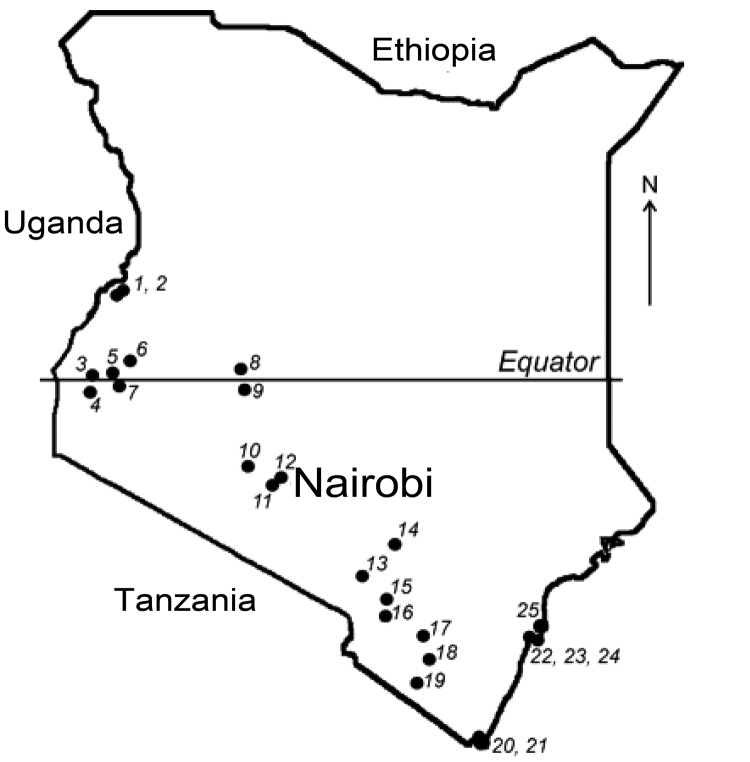
Field sites where bats were collected in Kenya. Numbers identify collection sites (*5*).

More than 500 bats were collected from 25 locations across Kenya (Figure 1). Sampling sites were chosen on the basis of available information about bat roosts and by using field observations of flying and foraging bats. The number of samples and the collection protocol were justified and approved by the National Museums of Kenya and the Kenyan Wildlife Service. Detailed information on the collection procedure has been described elsewhere (*5*). Briefly, bats were collected by using hand nets or collected manually in caves and human dwellings and were captured in mist nets around roosts or in locations of nocturnal foraging. Captured bats were anesthetized by an intramuscular injection of ketamine hydrochloride (0.05–0.1 mg/g bodyweight) and euthanized under sedation in compliance with the field protocol approved by CDC’s Institutional Animal Care and Use Committee. The bats were measured, and their sex and species were identified.

Species were identified phenotypically by using available field keys. Additionally, representative tissues of each collected species were submitted for confirmation to Guelph University (Guelph, Ontario, Canada), where partial sequences of the cytochrome oxidase gene were generated and compared with those available from the database of the Barcode of Life Data Systems (BOLD; www.barcodinglife.org). Because the taxonomy of African bats is under development and reference sequences for several species were unavailable in the BOLD database, the examined sequences were identified to the genus level only.

For microbiologic studies, selected organs and swabs were collected by using sterile plastic tubes. Serum was separated from the blood clots by centrifugation. All samples were transported on dry ice and stored at –80°C until testing.

### Culture

Preliminary attempts to culture *Bartonella* spp. from bat blood showed that the technique developed for isolation of these bacteria from rodent blood (*18*) is appropriate, with some minor modifications, for processing bat samples. Specifically, we used agar plates supplemented by a 10% addition of rabbit blood. Blood from bats was resuspended in brain–heart infusion (BHI) broth supplemented with 5% amphotericin B. The ratio between blood and BHI was determined to be 1:4. However, this ratio was difficult to adhere to for samples with a limited amount of blood (obtained from small bats). Although dilution of tested blood reduces our ability to detect bacteria in cases with a low level of bacteremia, this approach allowed us to reduce the likelihood that bacterial and fungal contaminants would overgrow the fastidious and slow-growing *Bartonella* spp. bacteria. Bacterial colonies were presumptively identified as *Bartonella* spp. on the basis of their morphology and later were confirmed by PCR amplification and sequencing of a specific fragment of the *gltA*. Subcultures of *Bartonella* spp. colonies from the original agar plate were streaked onto secondary agar plates, also supplemented by a 10% addition of rabbit blood, and, in case of confluent and pure cultures, harvested and stored in 10% glycerol. Agar plates inoculated with bat blood were incubated at 35°C in an aerobic atmosphere of 5% carbon dioxide for ≤30 days postinoculation, and plates with subcultures were incubated with the same conditions until sufficient growth was observed, usually 5–7 days.

### Amplification of the *gltA* Fragment

A heavy suspension of the microorganisms was heated for 10 min at 95°C followed by 1-min centrifugation at 8,000 rpm to precipitate the lysed cells. The supernant containing the genomic DNA was then moved to a clean centrifuge tube to be examined. PCR amplifications were performed in a 25-μL reaction mixture containing 5 μL 5× Green GoTaq PCR buffer (Promega, Madison, WI, USA), 0.4 μmol of each primer, 200 μM each dNTP, 1 U Taq DNA polymerase (Promega), and ≈20 ng of template DNA. Two oligonucleotides, BhCS781.p (5′-GGGGACCAGCTCATGGTGG-3′) and BhCS1137.n (5′-AATGCAAAAAGAACAGTAAACA-3′) were used as PCR primers to generate a 379-bp amplicon of the *Bartonella gltA* gene. Positive and negative controls were included with each PCR to evaluate the presence of appropriately sized amplicons and contamination, respectively. Each PCR was performed in a PTC 200 Peltier thermal cycler (MJ Research, Inc., Waltham, MA, USA) or in an Eppendorf Mastercycler Gradient (Eppendorf, Westbury, NY, USA). PCR products were separated by 1.5% agarose gel electrophoresis and visualized by ethidium bromide staining.

### Sequencing and Analysis of DNA

PCR products of correct size were purified with the QIAquick PCR Purification Kit (QIAGEN, Valencia, CA, USA) according to manufacturer’s instructions and sequenced in both directions by using an Applied Biosystems Model 3130 Genetic Analyzer (Applied Biosystems, Foster City, CA, USA). Sequencing reactions were performed in a PTC 200 Peltier Thermal cycler by using the same primers as the initial PCR with a concentration of 1.6 µM. Sequences were analyzed by using Lasergene (DNASTAR, Madison, WI, USA) sequence analysis software to determine consensus of sequences for the amplified region of the *gltA* gene. The Clustal V program within MegAlign (DNASTAR) was used to align and compare homologous *Bartonella* spp. *gltA* sequences obtained from bat samples and from the GenBank database.

## Results

### *Bartonella* spp. Cultures

Culturing from bat blood pellets, especially from small-sized bats, presented a challenge because of the limited sample volume and the potential for contamination with other bacteria and fungi, problems hard to avoid during field sampling. Consequently, of >500 processed blood samples, the presence or absence of *Bartonella* spp. cultures have been conclusively evaluated in samples from 331 bats of 13 species from 9 genera. Further estimation of the infection rate was determined exclusively on the basis of the specimens from these 331 animals. The time required for growth of *Bartonella* spp. colonies on agar greatly varied from 3 days, observed for several samples from *Triaenops persicus* bats to 28 days in one of the samples from *Rousettus aegyptiacus* bats.

### *Bartonella* spp. Prevalence

*Bartonella* isolates were cultured from the blood of 30.2% (106/331) of the bats tested. All isolates were confirmed genetically by amplification and sequence of the *gltA*. Prevalence of bats positive for *Bartonella* spp. by culture was as follows: *Eidolon helvum* (straw-colored fruit bat), 23/88 (26.1%); *R. aegyptiacus* (Egyptian fruit bat), 22/105 (21.0%); *Coleura afra* (African sheath-tailed bat), 4/9 (44.4%); *T. persicus* (Persian trident bat), 7/8 (87.5%); *Hipposideros commersoni* (giant leaf-nosed bat), 1/4 (25.0%); and *Miniopterus* spp. (long-fingered bats), 49/87 (56.3%) (Table). *Miniopterus* spp. and *T. persicus* bats were at significantly higher risk (p<0.0001 and p<0.001, respectively) for being infected with *Bartonella* spp. when each was compared with all other bats, and *R. aegyptiacus* and *Epomophorus* spp. bats were at significantly lower risk for infection (p<0.01 for both) (Table).

**Table:** Prevalence of culture-positive test results for *Bartonella* spp. among bat species, Kenya*

Bat species	No. tested	No. (%) positive	Relative risk	p value
*Miniopterus* spp.	87	49 (56)	2.41	<0.0001
*Eidolon helvum*	88	23 (26)	NS	
*Rousettus aegyptiacus*	105	22 (21)	0.6	<0.01
*Coleura afra*	9	4 (44)	NS	
*Triaenops persicus*	8	7 (88)	2.85	<0.001
*Hipposideros commersoni*	4	1 (25)	NS	
*Epomophorus* spp.	23	0	Not defined†	<0.01
*Rhinolophus* spp.	6	0	Not defined†	
*Chaerephon* sp.	1	0	Not defined†	

*Relative risk was calculated by comparing proportions positive for each bat species compared with that for all other bats. NS, not statistically significant. †Relative risk is <1 but is not defined because of 0 in 1 of the cells.

### Genetic Heterogeneity and Sequence Clustering

Sequence analyses of DNA from the 94 *Bartonella* isolates obtained from bats revealed 58 *gltA* genotypes (unique sequence variants with ≥1 nucleotide difference) that represented 11 genogroups with a sequence identity value of >96% between genotypes within each group, as proposed by La Scola et al. (*28*), as a cutoff for this specific gene fragment for species definition within the *Bartonella* genus. The 11 novel *gltA* sequences representing each genogroup were submitted to GenBank and assigned accession nos. HM363764–363768 and HM545136–545141.

### Association of *Bartonella* Genotypes with Particular Bat Species

**Figure 2:**
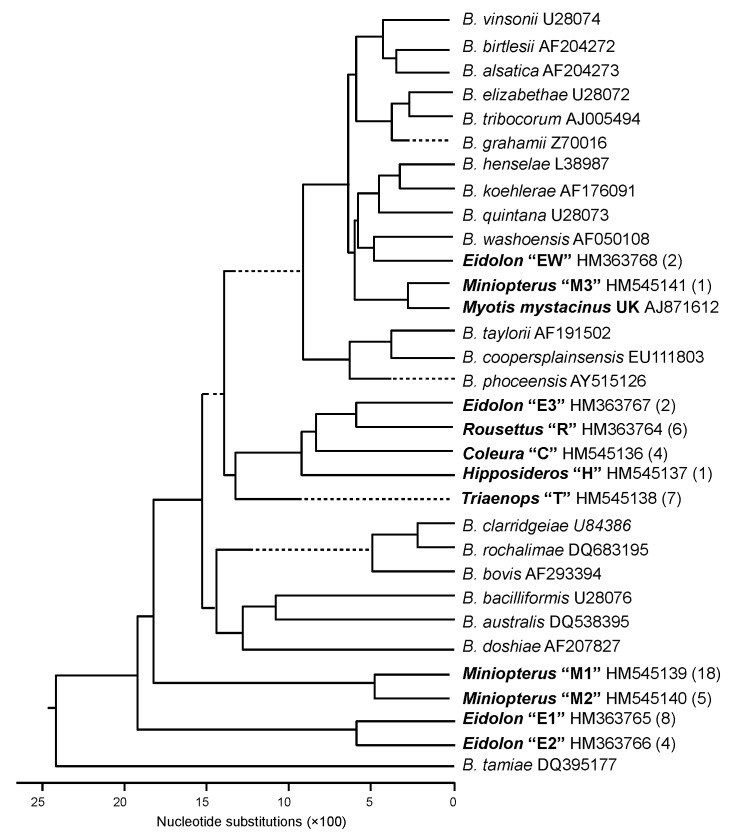
Phylogenetic relations among the citrate synthase sequences of *Bartonella* spp. genotypes detected in bats from Kenya and previously described *Bartonella* spp. The phylogenetic tree was constructed by the neighbor-joining method. Each *Bartonella* spp. genogroup detected in bats was provided with the Latin name of the bat genus from which the *Bartonella* strains were obtained (**boldface**), the proposed name of genogroup (quotation marks), the GenBank accession number, and the number of genotypes assigned to the genogroup (parentheses).

All 15 *Bartonella* spp. *gltA* sequences obtained from *R. aegyptiacus* bats were similar to each other (>96%) and distant from all sequences found in other bat species (<91% identity) and previously described *Bartonella* species (<84% identity). Six unique genetic variants identified in *R. aegyptiacus* bats were clustered in a monophyletic genogroup, marked as R in Figure 2.

The level of *gltA* sequence identity between 16 *Bartonella* genotypes identified in *E. helvum* bats varied greatly, ranging from 78.6%–100%. Eight genotypes (sequence variants) were clustered around 4 clades (genogroups), which are marked as E1, E2, E3, and EW in Figure 2. Intergroup identity values ranged from 78.6%–87.2%, much higher values than the sufficient criteria proposed for species demarcation within the *Bartonella* genus (*28*).

All 4 *gltA* genotypes of *Bartonella* spp. identified in *C. afra* bats differed slightly from one another but clustered tightly within genogroup C with a sequence identity range of 98.2%–99.7%, compared with a <90% identity between these sequences and *Bartonella* strains found in other bat species or other sources. Similarly, all 7 *gltA* sequences identified in *T. persicus* bats formed a monophyletic genogroup, marked as T in Figure 2, with the identity range of 98.2%–99.7% within the group and <90% identity to any *Bartonella* sequence outside the group. The only isolate obtained from *H. commersoni* bats also differed from all described *Bartonella* strains or sequences (identity <85%).

More complex phylogeny was observed when isolates obtained from bats of the genus *Miniopterus* were compared by using a similarity between the *gltA* sequences. In total, 51 *Bartonella* spp. *gltA* sequences, obtained from 3 identified *Miniopterus* spp. (*M. africanus*, *M. minor*, and *M. natalensis*) and from *Miniopterus* spp. bats that have not been identified at the species level, were analyzed. Among the 51 bats, 24 unique *gltA* genotypes represented at least 3 genogroups with a level of divergence ranging from 6.6%–18.5%, higher than has been recommended previously for differentiation of *Bartonella* spp (*28*). The first genogroup, M1, was composed of 30 sequences obtained from *M. minor* bats, 1 sequence from *M. natalensis* bats, and 1 sequence from an unidentified species within the genus *Miniopterus.* The second genogroup, M2, consisted of 5 sequences from *M. minor* bats, 2 sequences from *M. africanus* bats, 1 sequence from *M. natalensis* bats, and 2 sequences from an unidentified species of *Miniopterus* spp. bats. Although the second genogroup looked more diverse, an insufficient number of isolates were available to describe an association of specific *Bartonella* spp. lineages to certain *Miniopterus* spp. bats. In addition, 1 genotype, M3, from *M. natalensis* bats, was distant from both groups (identity <90%), but was relatively close (identity 94.2%) to the genotype identified in a whiskered bat (*Myotis mystacinus*) from the United Kingdom (*11*).

## Discussion

This investigation has resulted in the identification and characterization of *Bartonella* strains in bats and describes the prevalence and genetic characteristics of *Bartonella* spp. in bats in Africa. Detection of viable bacteria in a high proportion of bats in ≥6 bat species suggests that *Bartonella* spp. infection is highly prevalent in bat communities in eastern Africa. Some bat species tested negative for the bacteria; however, we had few samples from these species, making speculation difficult concerning whether those species are truly free from *Bartonella* spp. infection. However, we identified some bat species that were statistically more likely to be infected with *Bartonella* spp.

A high prevalence of *Bartonella* spp. in bats, ranging from 21%–88% in various species, is especially surprising considering the life spans of bats. Numerous investigations have shown that prevalence of *Bartonella* spp. infection can reach high rates in rodent populations, but rodents usually live no longer than 1–2 years, whereas bats can live for >20 years (*6*). Explaining such high prevalence is difficult without assuming persistent infection. This hypothesis was not confirmed for some rodent species, specifically for cotton rats; a high prevalence of *Bartonella* spp. in that population could be explained by replacement of diverse *Bartonella* strains sequentially colonizing an individual rat rather than by a long-term bacteremia (*29*). This scenario is unlikely in bats because they have much longer lives than rodents.

The lifestyle of bats, such as the colonial structure of their populations, close physical contact, aggressive interactions, and typically heavy ectoparasite infestations also might contribute to the frequent transmission of *Bartonella* spp. among individual animals. All *Bartonella* spp. are widely regarded as vector-transmitted agents, and diverse arthropods, such as sandflies, lice, fleas, ticks, and mites, have been implicated as potential vectors (*30*). Bats carry a wide range of ectoparasites, including bat flies, fleas, soft ticks, and mites, some of which are highly specific to bats (*31*). Limited information is available to suggest that alternative mechanisms beyond vector transmission may be responsible for the spread of *Bartonella* spp. infections among animals. For example, identification of viable *Bartonella* spp. bacteria in the blood of cotton rat embryos and neonates indicated a possibility of vertical transmission from a pregnant female to offspring (*32*). Detection of *Bartonella* spp. DNA in the saliva of dogs suggests a potential possibility of transmission through biting (*33*), and transmission of *Bartonella henselae* from cats to humans by cat scratch is well documented (*24*).

Comparative analyses of the *gltA* sequences obtained from *Bartonella* spp. cultures showed that bats in Kenya harbor a diverse assemblage of *Bartonella* strains, some of which appear to represent distinct species. Although we used only a portion of the citrate synthase gene for phylogenetic analysis, this gene has been shown to be a reliable tool for distinguishing between closely related *Bartonella* genotypes (*28**,**34*). By using this gene, we were able to compare the variety of *Bartonella* genotypes isolated from bats with homologous sequences of *Bartonella* strains found in other mammals. Finding considerable sequence diversity is typical for different species of Bartonella, although more characteristics are needed to describe novel *Bartonella* species.

Evidence for cospeciation of *Bartonella* spp. with natural hosts varies among studies and around the world. A strong association exists between specific *Bartonella* species and their mammalian hosts, whereas some observations have indicated that 1 species of *Bartonella* can infect a variety of rodent species at a given site in Europe (*35*). Our investigation indicated a definite host-specificity for *Bartonella* strains in bat species. All isolates obtained from *R. aegyptiacus, C. afra,* and *T. persicus* bats clearly belonged to the specific *Bartonella* spp. group found exclusively in the particular bat species. By contrast, straw-colored fruit bats (*E. helvum*) and long-fingered bats (*Miniopterus* spp.) harbored strains clustered around 3 and 4 different groups of *Bartonella* spp., respectively, based on their *gltA* identity. Nevertheless, all strains of *Bartonella* spp. recovered from *E. helvum* bats were typical for this species of bats only. This pattern of cospeciation resembles the *Bartonella* spp.–host relations observed in cotton rats (*18*). Similarly, the *gltA* sequences from all strains obtained from *Miniopterus* spp. bats have not been found in bats of other bat genera. More investigations of *Bartonella* spp. in diverse bat species are required to test this hypothesis.

No evidence is available to suggest whether novel strains of *Bartonella* spp. found in bats from Kenya cause human illness. However, relevant surveillance in Kenya and other African countries has not been implemented. The significance of African bats in public and veterinary health is not understood because of a lack of surveillance. Bats are known as principal reservoir hosts of lyssaviruses. In Africa, these include Lagos bat virus, which circulates in pteropid bats (including *E. helvum* and *R. aegyptiacus*), and Duvenhage virus, which circulates in insectivorous bats (although specific reservoirs have not been established). In addition, Shimoni bat virus was identified recently in an insectivorous bat *Hipposideros commersoni* (*32*). Furthermore, multiple species of African bats have been shown to harbor coronaviruses (*33*). Nipah virus was identified in straw-colored fruit bats *E. helvum* (*34*), and Marburg virus was identified in tomb bats *R. aegyptiacus* (*35*). Circulation patterns of these agents in bat populations have not been sufficiently studied.

Characterization of the isolates obtained from bats and comparison with those obtained from human cases associated with *Bartonella* spp. of unknown origin (e.g., *B. tamiae*) can be helpful in the search for potential reservoirs. The reagents prepared from bat isolates of *Bartonella* spp. can be used for serologic surveys conducted in high-risk areas of the world. Application of antigens produced from bat-derived strains is especially relevant to serologic investigations of cases of infectious disease of unknown origin among persons occupationally exposed to bats. In addition, cocirculation of *Bartonella* spp. with other pathogens in a bat population can affect the bat’s ecology and pathobiology. These aspects need further investigation.
